# Recent advances in the genetics of idiopathic pulmonary fibrosis

**DOI:** 10.1097/MCP.0000000000000989

**Published:** 2023-07-06

**Authors:** Paolo Spagnolo, Joyce S Lee

**Affiliations:** a Respiratory Disease Unit, Department of Cardiac Thoracic, Vascular Sciences and Public Health, University of Padova, Padova, Italy; b University of Colorado Denver, Anschutz Medical Campus, Aurora, Colorado, USA

**Keywords:** genetics, idiopathic pulmonary fibrosis, mutations, pathogenesis, telomeres

## Abstract

**Purpose of review:**

Genetics contributes substantially to the susceptibility to idiopathic pulmonary fibrosis (IPF). Genetic studies in sporadic and familial disease have identified several IPF-associated variants, mainly in telomere-related and surfactant protein genes.

Here, we review the most recent literature on genetics of IPF and discuss how it may contribute to disease pathogenesis.

**Recent findings:**

Recent studies implicate genes involved in telomere maintenance, host defence, cell growth, mammalian target of rapamycin signalling, cell–cell adhesion, regulation of TGF-β signalling and spindle assembly as biological processes involved in the pathogenesis of IPF. Both common and rare genetic variants contribute to the overall risk of IPF; however, while common variants (i.e. polymorphisms) account for most of the heritability of sporadic disease, rare variants (i.e. mutations), mainly in telomere-related genes, are the main contributors to the heritability of familial disease. Genetic factors are likely to also influence disease behaviour and prognosis. Finally, recent data suggest that IPF shares genetic associations – and probably some pathogenetic mechanisms – with other fibrotic lung diseases.

**Summary:**

Common and rare genetic variants are associated with susceptibility and prognosis of IPF. However, many of the reported variants fall in noncoding regions of the genome and their relevance to disease pathobiology remains to be elucidated.

## INTRODUCTION

Idiopathic pulmonary fibrosis (IPF) is an inexorably progressive interstitial lung disease (ILD) of unknown origin with a poor prognosis and limited response to treatment. Indeed, currently available therapies (i.e. nintedanib and pirfenidone) target only a handful of fibrogenic pathways that are involved in disease pathogenesis [1^▪^]. Genetic variants contribute substantially to IPF risk, although common variants (i.e. polymorphisms) account for most of the heritability of sporadic disease while rare variants (i.e. mutations), mainly in telomere-related genes (TRG), are the main contributors to the heritability of familial disease [2,3]. However, a recent meta-analysis concluded that IPF is highly polygenic with a significant number of associated variants yet to be identified [4]. Only a small minority of carriers of common risk variants develop pulmonary fibrosis, suggesting that additional environmental and/or genetic factors are needed for the final phenotype to emerge. 

**Box 1:**
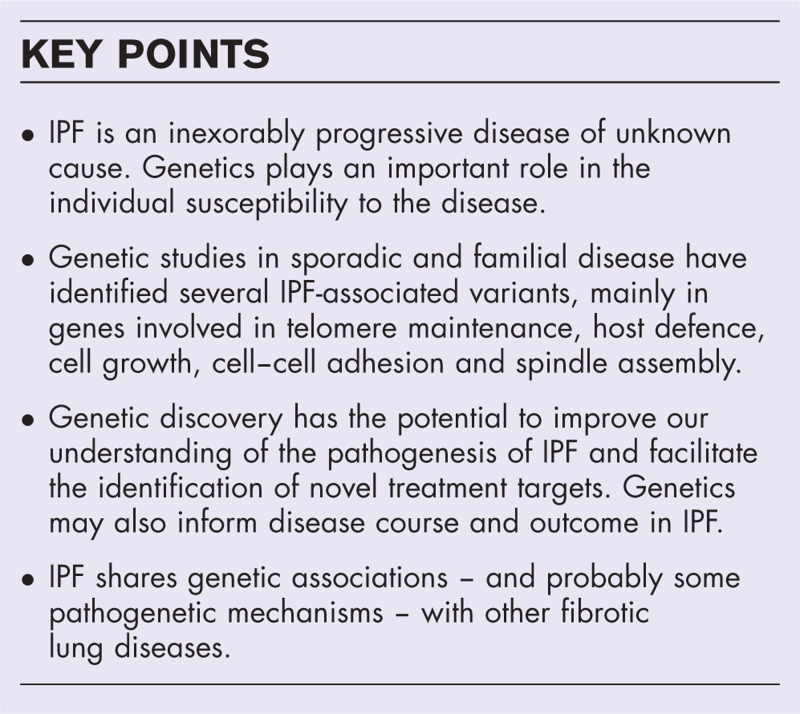
no caption available

In this review, we summarize the most relevant genetic studies of IPF published in the last 2 years (Table 1) and discuss how genetic discovery may improve our knowledge of disease pathogenesis and facilitate the identification of novel treatment targets. We also explore how genetics may inform disease course and outcome in IPF as well as genetic links between IPF and other fibrotic ILDs.

**Table 1:** Selected newly identified genetic variants in idiopathic pulmonary fibrosis

Gene	Locus	Variant	Location	Study methodology	Gene function	Reference
IL9RP3 pseudogene	16p13.3	rs367849850	Exon	WGS	Unknown	[6]
KIF15	3p21.31	rs74341405	Intron	WES	Mitotic spindle assembly	[10^▪▪^]
KNL1	15q15.1	Sentinel variant	Intron	GWAS	Mitotic spindle-assembly checkpoint signalling; correct chromosome alignment	[7]
NPRL3	16p13.3	Sentinel variant	Intron	GWAS	Regulator of mTORC1 signalling pathway	[7]
STMN3	20q13.33	Sentinel variant	Intron	GWAS	Microtubule-destabilizing activity	[7]
RTEL1	20q13.33	Sentinel variant	Intron	GWAS	Telomere length regulation, DNA repair, maintenance of genomic stability	[7]
Unknown	10q25.1	Intergenic	Intergenic	GWAS	–	[7]
TERT	5p15.33	rs199422297	Exon	WGS	Enzymatic component of telomerase	[12^▪▪^]
RTEL1	20q13.33	rs373740199	Exon	WGS	Telomere length regulation, DNA repair, maintenance of genomic stability	[12^▪▪^]
SPDL1	5q35.1	rs116483731	Exon	WGS	Mitotic spindle formation and chromosome segregation	[12^▪▪^]
KIF15	3p21.31	rs138043992	Exon	Global Biobank Meta-analysis	Microtubule binding and motor activity	[15]
POT1	7q31.33	NA (c.776 T>C)	Exon	Exome sequencing	Telomere maintenance	[18]

mTORC1, mammalian target of rapamycin complex 1; WES, whole-exome sequencing; WGS, whole exome sequencing.

## GENETIC SUSCEPTIBILITY TO IDIOPATHIC PULMONARY FIBROSIS

To date, several genetic loci have been associated with IPF. These associations implicate genes involved in telomere maintenance, host defence, cell–cell adhesion, regulation of TGF-β signalling and spindle assembly as biological processes involved in disease pathogenesis [2,3,5]. Donoghue *et al.*[6] performed a whole genome sequencing (WGS) in a large cohort of IPF patients (*n* = 1638) and matched controls (*n* = 7947). A novel genome-wide significant association with increased risk for IPF was identified at 16p13.3 in the subtelomeric region within the pseudogene *IL9RP3*, which maps ∼70 kb from *NPRL3*, another IPF-associated gene [7]. Intriguingly, carriage of the *IL9RP3* variant is associated with longer telomeres, which is in contrast with the established inverse correlation between telomere length and IPF risk [8,9]. Whether the 16p signal confers susceptibility to IPF through a mechanism independent of telomere length remains to be elucidated.

Zhang *et al.*[10^▪▪^] have performed the largest IPF whole-exome sequencing (WES) study to date. Novel deleterious variants were identified within *KIF15*, a kinesin involved in spindle separation during mitosis, nearby a common intronic variant previously associated with IPF [4]. These associations were identified in the derivation cohort (1725 cases and 23 509 controls) and confirmed in meta-analyses of the discovery and replication cohorts, which totalled 2966 cases and 29 817 controls. Significantly, in-vivo studies demonstrated that carriage of *KIF15* variants leads to reduced gene expression and rates of cell growth, suggesting a link between telomere-independent pathways of cell proliferation and susceptibility to IPF.

Following a genome-wide association study (GWAS) consisting of three and two independent datasets as discovery and replications cohorts, respectively [4], Allen *et al.*[7] performed a meta-analysis of genome-wide data from all five datasets (4125 cases, 20 464 controls and 7554 248 variants). Five novel association signals were identified within *KNL1*, *NPRL3*, *STMN3*, *RTEL1* and in an intergenic region mapping to 10q25.1, which implicates mammalian target of rapamycin signalling, telomere maintenance and spindle assembly genes in susceptibility to IPF.

### Genetic studies of rare variants

Rare genetic variants of protein-coding genes have been associated with IPF [11], but the extent to which they contribute to disease risk remains unknown. Peljto *et al.*[12^▪▪^] addressed this question by sequencing 2180 IPF cases and found that rare variants within *TERT* and *RTEL1*, one mutation in each of the two genes, are largely responsible for the observed association with IPF. Notably, none of the previously reported associations with rare variants was confirmed. This study also found that well established common variants contribute the most to the overall risk of IPF, with the single nucleotide polymorphism-heritability of IPF being 32%.

Variants in genes related to surfactant biology occur in 1–3% of IPF patients often in association with lung cancer [13]. Sutton *et al.*[14] evaluated the prevalence of rare variants in five surfactant-related genes, *SFTPA1*, *SFTPA2*, *SFTPC*, *ABCA3* and *NKX2-1* in 431 patients with IPF. Functionally deleterious variants were identified in 1.3% of patients regardless of a history of lung cancer or a family history of IPF. Notably, patients carrying a *SFTPA2* mutation were younger, had longer telomeres and were more likely to have a chest computed tomography inconsistent with usual interstitial pneumonia compared with the remaining patients.

### Genetic studies of multiple ancestries

Genetic studies of IPF have mostly included patients of European ancestry; therefore, common and rare genetic variants have been understudied (and are likely to be underreported) in IPF patients of non-European ancestry. Partanen *et al.*[15] performed the first multiancestry meta-analysis including 11 000 patients and 1.4 million controls from six ancestries and 13 biobanks worldwide. Beyond confirming most of the previous associations with IPF, the authors identified seven novel genome-wide significant loci, including a putative functional variant within *KIF15*. Significantly, the authors calculated that only one of the novel variants would have been identified had the analysis been limited to individuals of European ancestry. A sex-stratified meta-analysis revealed a 1.6-fold larger effect of the *MUC5B* rs35705950 locus in male patients, but this is likely due to case ascertainment differences across biobanks. Zhang *et al.*[16^▪^] performed the first genome-wide study of rare, deleterious (i.e. missense and protein-truncating) variants in non-European IPF patients (*n* = 241) and controls (*n* = 12 509) and found an excess of *TERT* variants exceeding genome-wide significance in the Latino subgroup. Conversely, no enrichment of *PARN*, *RTEL1* and *KIF15* variants was observed.

### Telomere shortening and dysfunction

In IPF patients, short telomeres – defined as telomere length at or below the 10th percentile for age – are a common finding regardless of the carriage of variants in TRG [8,9]. However, it is unclear whether short telomeres observed later in life are inherited or a result of accelerated attrition. Salisbury *et al.* found that unaffected first-degree relatives of patients with familial IPF have shorter than expected telomeres, regardless of personal or familial status of the telomerase mutation carrier. Further, increased telomere attrition contributed to reduced telomere length among individuals at risk for familial pulmonary fibrosis (FPF) [17].

Shelterin is a six-protein complex that protects chromosome ends. POT1-TPP1 is one such protein, which acts by regulating both telomere capping and length. A novel heterozygous mutation within *POT1* (L259S) has recently been identified by exon sequencing in a family that displayed short telomeres and genetic anticipation [18]. Fibroblasts isolated from patients carrying this mutation showed significant telomere loss and telomere dysfunction, suggesting that *POT1* L259S may be pathogenic in IPF – at least in a subset of patients – by leading to accelerated senescence and growth defects. Zhang *et al.*[19] performed WGS on 949 patients with IPF or FPF to determine rare and common variant genotypes, estimate telomere length and assess the association between genetic variants with clinically relevant disease outcomes. Rare deleterious variants were carried by 14% of the total cohort, mainly by patients with a family history of the disease were mostly found in TRG and were associated with shorter telomere lengths and disease progression.

### Familial pulmonary fibrosis

**FIGURE 1:**
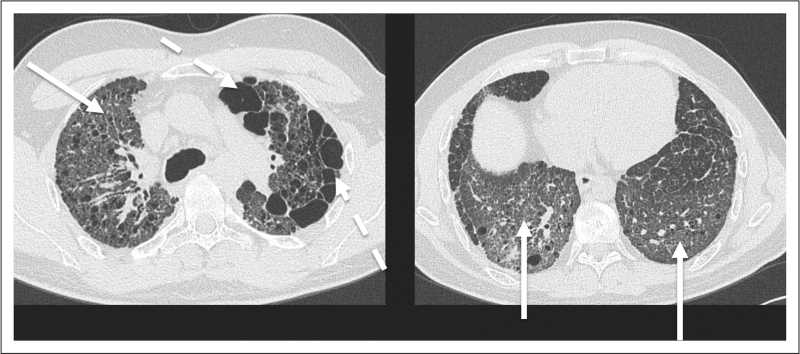
A 41-year-old man with familial pulmonary fibrosis listed for lung transplantation. High-resolution computed tomography shows ground glass opacity (continuous arrows), and paraseptal emphysema (dashed arrows). Both abnormalities predominate in the upper zones. This patient was found to carry a heterozygous *TERC* mutation.

Up to 20% of ILD cases are reported to be familial (Fig. 1) [20–22]. Liu *et al.*[23^▪▪^] performed a comprehensive evaluation for rare genetic variants by WES and/or candidate gene sequencing in a large cohort of FPF kindreds (*n* = 569). They found that rare variants, mainly in TRG, account for 14.9–23.4% of FPF genetic risk, with pathways enriched for rare variant-containing genes, including focal adhesion and mitochondrial complex I assembly. These findings suggest that disrupted mitochondrial homeostasis and bioenergetics may contribute to fibrotic remodelling of the lung [24]. In addition, rare-variant containing genes were overrepresented in type II alveolar epithelial cells, smooth muscle cells and endothelial cells, indicating a role for these cell types in disease pathogenesis. Notably, no new rare variant-containing gene shared across multiple kindreds was identified, suggesting that, in most FPF kindreds, genetic risk is mediated by a multitude of genetic variants involving several (rather than a few well defined) profibrotic genes and pathways. However, larger FPF kindreds might reveal novel disease-associated genes.

## CONTRIBUTION OF GENETIC FACTORS TO IDIOPATHIC PULMONARY FIBROSIS PATHOGENESIS

A gain-of-function promoter polymorphism within *MUC5B* (rs35705950-T), which is carried by approximately 50% of IPF patients, is the risk variant with the largest effect for both sporadic and familial disease [25]. Although the minor T allele is associated with increased MUC5B expression, genotype alone does not explain the substantial variability in MUC5B levels observed in IPF [26]. Yang *et al.*[27] originally identified an IPF endotype enriched for cilium-associated genes and characterized by high expression of MUC5B. Following up on this study, Ghosh *et al.*[28] used lung tissue RNA sequencing to recreate a cilium and noncilium enriched endotype with the aim to assess the relationship between rs35705950 genotype and these two endotypes. They confirmed that MUC5B expression is higher in the cilium endotype. However, the association between rs35705950 genotype and MUC5B expression (i.e. GG > GT > TT) was only seen in the noncilium endotype, demonstrating that the *MUC5B* promoter variant accounts only partially for the variability in MUC5B expression between the cilium and noncilium endotype. Finally, the two endotypes were clinically similar but differed in estimated cell type proportions, with the cilium endotype displaying a lower proportion of macrophages and type II alveolar epithelial cells and a higher proportion of club and basal cells. Whether and to what extent these represent pathogenetic differences remains to be elucidated.

Borie *et al.*[29^▪▪^] focused on genetic variants within 10 genetic loci associated with IPF and identified 24 expression quantitative trait loci (eQTL) in IPF cases and 27 eQTLs in nondiseased controls. Importantly, they found that the minor variants of *MUC5B* rs35705950 and *DSP* rs2076295 altered the expression and DNA methylation of the respective gene in both cases and controls. In addition, these findings demonstrate that the expression of MUC5B and DSP is regulated by genetic and epigenetic factors.

In complex diseases like IPF, wherein each associated gene/locus exerts a relatively small effect, it is either the cumulative effect of multiple risk variants or the interaction between genes and environment that contributes the most to disease susceptibility [1^▪^].

### The effects of air pollutants and genetic susceptibility to idiopathic pulmonary fibrosis risk

Accumulating epidemiologic and translational data support a relationship between air pollution exposure and the development of pulmonary fibrosis [30,31]. Cui *et al.*[32^▪^] used data from the UK Biobank, a large-scale prospective study, to assess the effects of genetic susceptibility on the association between long-term exposures to air pollutants and IPF. To this end, the authors quantified annual average air pollution concentrations for NO_2_, nitrogen oxides (NO_x_), fine particulate matter with diameter less than 2.5 μm (PM_2.5_) and PM_10_ by a land-use regression model and constructed a polygenic risk score (PRS) using 13 polymorphisms previously associated with IPF. They documented 1380 incident cases of IPF over 5028 205 person-years of follow-up, with the risk of IPF being higher among individuals who were older, males, smokers, overweight and obese and with a higher PRS. Long-term exposure to NO_2_, NO_x_ and PM_2.5_ was associated with an increased risk of incident IPF; however, the highest risk of incident IPF was observed among individuals with a high PRS and high air pollution exposure. In addition, the authors observed positive additive interactions between NO_2_, NO_x_ and PM_2.5_ and PRS on the risk of IPF, suggesting a synergistic effect. Taken together, these data suggest that improving air quality may reduce the incidence of IPF, especially in individuals with high genetic risk.

## GENETIC FACTORS MAY DRIVE DISEASE BEHAVIOUR AND PROGNOSIS

In IPF, predicting disease behaviour remains challenging, with the majority of patients declining slowly over time, while others experience more rapid progression or even acute exacerbation [33]. Allen *et al.*[34^▪▪^] performed a genome-wide meta-analysis of over 7 million common variants with the aim to identify predictors of functional decline in IPF. The study included 1329 patients with 5216 measures for the forced vital capacity (FVC) analysis and 975 patients with 3361 measures for the diffusing capacity of the lung for carbon monoxide (DL_CO_) analysis. Surprisingly, only one variant [located in an antisense RNA gene for protein Kinase N2 (PKN2)] was associated with FVC decline. PKN2 is a Rho and Rac effector protein involved, among others, in cell cycle progression, cell migration and cell adhesion [35]. The finding that this variant is not one of the well-established risk factors for IPF suggests that genetic factors conferring susceptibility to IPF may be different from those driving the trajectory of lung function decline. This concept applies also to *MUC5B* rs35705950 wherein the minor T allele is associated with both risk of IPF and a more favourable prognosis [36,37]. The study by Allen *et al.*, however, did not assess the effect on FVC decline of rare coding variants, such as those related to telomere maintenance and function, and included only IPF patients of European ancestry. Therefore, its findings are not generalizable to all ethnicities.

A functional polymorphism within Toll-like 3 (*TLR3*; L412F) has been associated with accelerated FVC decline and shorter survival in patients with IPF [38]. McElroy *et al.*[39] investigated the effect of *TLR3* L412F on the lung microbiome and antibacterial TLR activity of primary lung fibroblasts from patients with IPF. They found that *TLR3* L412F alters the bacterial load and composition of the IPF lung microbiome and attenuates the response of IPF lung fibroblasts to bacterial or viral infections, either alone or in combination. Notably, carriage of the 412F-variant was also associated with an increased risk of death by acute exacerbation, suggesting a potential mechanism by which dysfunctional TLR3 predisposes to viral- and bacterial-mediated acute exacerbation in IPF.

Oldham *et al.*[40^▪▪^] performed a two-stage GWAS of IPF survival that included two independent cohorts of 1481 cases in stage 1 and 397 cases in stage 2. They identified four variants associated with differential transplant-free survival (TFS), including one in an intron of proprotein convertase subtilisin/kexin type 6 (*PCSK6*) that reached genome-wide significance. PCSK6 was highly expressed in IPF lung tissue, most highly in the airway epithelium; in addition, downstream analysis demonstrated that PCSK6 lung staining intensity, peripheral blood gene expression and circulating plasma concentration negatively correlated with TFS, suggesting an important role for PCSK6 as mediator of IPF progression. However, because of its rarity, the *PCSK6* variant identified in this study is unlikely to fully explain subsequent gene expression and protein findings.

IPF has become the most common indication for lung transplant worldwide. Alder *et al.*[41] performed genetic evaluation of 431 IPF patients (using WGS in 426 individuals and targeted sequencing in five), including 149 who underwent lung transplantation. Compared with nontransplanted patients, those transplanted were significantly younger (60 vs. 70 years), twice as likely to carry TRG rare variants (24 vs. 12%) and had shorter telomeres, with 85% of them displaying telomeres below the age-adjusted mean. However, posttransplant survival was similar regardless of carriage of mutations in TRG and telomere length, indicating that IPF patients with telomere-mediated disease should not be excluded *a priori* from lung transplantation.

## COMMONALITY BETWEEN IDIOPATHIC PULMONARY FIBROSIS AND OTHER FIBROTIC LUNG DISEASES

A subset of patients with hypersensitivity pneumonitis develops pulmonary fibrosis with clinical features and outcome similar to those of IPF [42]. Although this phenotype is believed to result from persistent exposure to the triggering antigen, genetic factors may also play a role. Furusawa *et al.*[43] conducted a case–control study of 226 hypersensitivity pneumonitis patients and 1355 controls to investigate the distribution of 10 IPF-associated polymorphisms. Six common variants were significantly associated with hypersensitivity pneumonitis, with *MUC5B* rs35705950 showing the strongest association with both hypersensitivity pneumonitis as a whole [odds ratio (OR); 2.11, *P* = 1.74 × 10^−6^] and fibrotic hypersensitivity pneumonitis (OR; 2.32, *P* = 2.61 × 10^−6^). *MUC5B*, *TERC* and *IVD* remained statistically significant after including all variants in a single model of fibrotic hypersensitivity pneumonitis. Taken together, these findings support the hypothesis that IPF and fibrotic hypersensitivity pneumonitis may share a common pathogenesis.

Pulmonary fibrosis may complicate a significant minority of cases of COVID-19 pneumonia [44]. Although the exact prevalence and risk factors of this severe sequela are yet to be established, individuals with ILD are at higher risk of death from COVID-19 [45]. In the largest IPF and COVID-19 GWAS to date, Allen *et al.*[46] explored whether any of the previously reported IPF risk variants were also associated with COVID-19. Shared genetic signals between IPF and severe COVID-19 were identified near *MUC5B*, *DPP9* and *ATP11A*, although the signals at *MUC5B* and *ATP11A* had opposite effects on the risk for the two diseases.

## GENETIC SCREENING

Relatives of individuals with IPF are at increased risk of developing the disease [47]. Genetic screening may therefore detect the disease in a preclinical phase, thus serving as an important adjunct in clinical decision-making. However, there are no clinical guidelines on which tests to include, the age at which screening should be initiated and how often to repeat it. Most expert centres offer genetic testing to patients with FPF and their first-degree relatives [48]. Additional clinical scenarios in which genetic testing might be considered include patients with pulmonary fibrosis with personal or family history of telomeropathy (e.g. familial liver cirrhosis, aplastic anaemia, acute myelogenous leukaemia), unaffected family members of probands carrying pathogenic variants in disease-causing genes, and individuals younger than 50 years at disease onset [49]. When genetic testing is considered, genetic counsellors should also be involved. The integration of genetic testing into clinical practice remains challenging and at present should be limited to specialized centres and genetic professionals.

## CONCLUSION

In the last few years, our understanding of the contribution of genetic factors to IPF risk has improved significantly. Genetic studies of IPF have also provided relevant information to patient care by informing disease outcome and identifying potential therapeutic targets. However, as the majority of genetic associations fall in noncoding regions of the genome, how and to what extent genetic factors contribute to disease development and progression remains largely unknown. In addition, most genetic discoveries are restricted to common variants in IPF patients of European descent, but the availability of global biobanking has the potential to fill this gap by increasing sample size and ethnic diversity.

## Acknowledgements

*None.*

### Financial support and sponsorship

*None.*

### Conflicts of interest

*There are no conflicts of interest.*

## REFERENCES AND RECOMMENDED READING

Papers of particular interest, published within the annual period of review, have been highlighted as:

▪ of special interest
▪▪ of outstanding interest
